# Functional Metagenomics Reveals an Overlooked Diversity and Novel Features of Soil-Derived Bacterial Phosphatases and Phytases

**DOI:** 10.1128/mBio.01966-18

**Published:** 2019-01-29

**Authors:** Genis Andrés Castillo Villamizar, Heiko Nacke, Marc Boehning, Kristin Herz, Rolf Daniel

**Affiliations:** a Department of Genomic and Applied Microbiology and Göttingen Genomics Laboratory, Institute of Microbiology and Genetics, Georg-August University, Göttingen, Germany; b Línea tecnológica biocorrosión, Corporación para la investigación de la corrosión C.I.C. Piedecuesta, Santander, Colombia; Hebrew University of Jerusalem

**Keywords:** SNARE-associated domain, functional metagenomics, phosphatases, phytases, soil metagenome

## Abstract

Phosphatases, including phytases, play a major role in cell metabolism, phosphorus cycle, biotechnology, and pathogenic processes. Nevertheless, their discovery by functional metagenomics is challenging. Here, soil metagenomic libraries were successfully screened for genes encoding phosphatase activity. In this context, we report the largest number and diversity of phosphatase genes derived from functional metagenome analysis. Two of the detected gene products carry domains which have never been associated with phosphatase activity before. One of these domains, the SNARE-associated domain DedA, harbors a so-far-overlooked motif present in numerous bacterial SNARE-associated proteins. Our analysis revealed a previously unreported phytase activity of the alkaline phosphatase and sulfatase superfamily (cl23718) and of purple acid phosphatases from nonvegetal origin. This suggests that the classical concept comprising four classes of phytases should be modified and indicates high performance of our screening method for retrieving novel types of phosphatases/phytases hidden in metagenomes of complex environments.

## Abstract (precis)

Phosphorus (P) is a key element involved in numerous cellular processes and essential to meet global food demand. Phosphatases play a major role in cell metabolism and contribute to control the release of P from phosphorylated organic compounds, including phytate. Apart from the relationship with pathogenesis and the enormous economic relevance, phosphatases/phytases are also important for reduction of phosphorus pollution. Almost all known functional phosphatases/phytases are derived from cultured individual microorganisms. We demonstrate here for the first time the potential of functional metagenomics to exploit the phosphatase/phytase pools hidden in environmental soil samples. The recovered diversity of phosphatases/phytases comprises new types and proteins exhibiting largely unknown characteristics, demonstrating the potential of the screening method for retrieving novel target enzymes. The insights gained into the unknown diversity of genes involved in the P cycle highlight the power of function-based metagenomic screening strategies to study Earth’s phosphatase pools.

## INTRODUCTION

Within the last decades, advances in next-generation sequencing and metagenomic techniques have led to the discovery of new enzymes from metagenomes (1, 2). Novel lipases, esterases, proteases, and hydrogenases, among many others, have been identified (3, 4). Nevertheless, the majority of enzymes with high biological relevance are still almost exclusively recovered from cultured organisms (2). This is especially the case for phosphatases. Phosphatases have evolved across all living organisms and contribute to the regulation of diverse cellular functions (5, 6). A specific group of phosphatases named phytases can release phosphorus from phytic acid, which is one of the most important phosphorus reserves in plants and soils (7, 8).

Phosphorus (P) reserves are globally important, due to the enormous growth of the world population, and the ensuing demand for this macroelement. Large amounts of P are and will be required in order to fulfill the increasing world agroalimentary needs (9). However, global rock phosphorus reservoirs are currently being rapidly depleted, and the supplementation of P to animal feed and plant fertilizers has become more expensive during the last decades (10). Plant-based animal feeds often contain large amounts of phytate, which cannot be utilized by monogastric animals due to the lack of phytases (7, 11). As a consequence, P levels in soils and water bodies increase. This eutrophication causes for instance algal blooms in aquatic ecosystems, leading to deoxygenated areas disturbing the life of many species (12). To meet future requirements, minimize losses of P, and reduce the environmental impact, it is necessary to use P compounds more efficiently and develop economical recycling technologies. In this context, phosphatases/phytases have proved to be remarkably useful (13). These enzymes are currently used in agroindustry to minimize P losses and to improve the levels of bioavailable P (14). A more recently described role of the phytases is their involvement in pathogenicity causing tissue damage in humans, coordination of the virulence program in Dickeya dadantii, and mediation of plant infection by Candida albicans and *Xanthomonas*, respectively (5, 15, 16).

The diversity and potential of environmental phytases remain largely unexplored as so far almost all reported functionally characterized phytases were derived from cultured organisms, including plants, fungi, and bacteria. Based on their catalytic characteristics, four classes of phytases have been described: histidine acid phytase (HAPhy), β-propeller phytase (BPPhy), purple acid phytase (PAPhy), and protein tyrosine phytase (PTPhy). These enzymes are structurally and catalytically dissimilar (14, 17).

In this study, we use a function-based screening approach (18) to identify environmental phosphatases/phytases. By using soil metagenomes as a source, we were able to recover novel genes encoding phosphatases with phytase activity. Some of the recovered genes encode protein domains that were not associated with phosphatase activity before, and others represent new types or subtypes of phytases.

## RESULTS

### Phosphatase detection strategy.

The metagenomic libraries contained approximately 38,122 to 166,040 clones and were screened for candidates exhibiting phosphatase activity using plates with phytate as phosphorus source and BCIP as indicator (see Fig. S1 in the supplemental material). The quality of the libraries was controlled by determining the average insert sizes and the percentage of insert-bearing Escherichia coli clones. The average insert sizes of metagenomic DNA-containing plasmids ranged from 2.8 to 6.7 kb, and the frequency of clones carrying plasmid inserts was at least 89% (Table 1).

**TABLE 1:** Characteristics of the soil metagenomic libraries and designation of plasmids harbored by positive clones

Library^*a*^	No. of clones	Avg insert size (kb)	Insert frequency (%)	Estimated library size (Gb)	No. of positive clones/Gb	Plasmid(s) of positive clones (accession no.)
AEW1*	129,748	6.7	91	0.79	1.2	pLP01 (KY931670)
AEW5*	90,300	5.2	89	0.42	2.3	pLP02 (KY931671)
SEW2*	135,240	5.7	95	0.73	9.6	pLP10 (KY931677), pLP14 to pLP19 (KY931679 to KY931684)
SEW5*	166,040	4.0	95	0.63	1.6	pLP07 (KY931674)
SEW46	38,122	2.8	93	0.17	23.5	pLP03 (KY931672), pLP04 (KY931673), pLP08 (KY931675), pLP09 (KY931676)
HEW30	53,460	6.1	96	0.31	22.6	pLP13 (KY931678), pLP20 (KY931685), pLP24 to pLP28 (KY931686 to KY931690)

a AEW, metagenomic libraries derived from the Biodiversity Exploratory Schwäbische Alb; SEW, metagenomic libraries derived from the Biodiversity Exploratory Schorfheide-Chorin; HEW, metagenomic libraries derived from the Biodiversity Exploratory Hainich-Dün. *, previously generated libraries (39).

10.1128/mBio.01966-18.1 FIG S1 Sperber medium indicator plate containing the negative control (E. coli DH5α carrying pCR-XL-TOPO) and a typical positive E. coli clone (E. coli DH5α carrying plasmid pLP03). Download FIG S1, PDF file, 0.5 MB. Copyright © 2019 Castillo Villamizar et al. 2019 Castillo Villamizar et al. This content is distributed under the terms of the Creative Commons Attribution 4.0 International license.

We recovered 21 positive E. coli clones from functional screens carrying plasmids harboring one or more ORFs associated with known phosphatase genes and domains (designation of plasmids is given in Table 1). The entire inserts of the positive clones were sequenced and taxonomically classified, showing that in all cases the cloned environmental DNA is of bacterial origin. Most inserts of the positive clones were affiliated with *Terrabacteria*, *Proteobacteria*, and the PVC superphylum with seven, six, and four representatives, respectively. Within the *Terrabacteria* group, most of the inserts (4) were affiliated with *Actinobacteria* (Table S1).

10.1128/mBio.01966-18.5 TABLE S1 Taxonomic classification of inserts from the positive clones harboring phosphatase-related genes by using Kaiju 1.5.0. Download Table S1, PDF file, 0.04 MB. Copyright © 2019 Castillo Villamizar et al. 2019 Castillo Villamizar et al. This content is distributed under the terms of the Creative Commons Attribution 4.0 International license.

Thirty-one ORFs encoding putative gene products with similarity to known phosphatase enzymes were identified. Signal peptides were detected for 12 of them. The deduced gene products comprised 214 to 819 amino acids with calculated molecular masses ranging from 12 to 65.5 kDa and amino acid sequence identities to the closest known phosphatases ranging from 25% (Pho14B) to 83% (Pho13) over the full-length protein (Table 2).

**TABLE 2:** Gene products encoded by genes associated with phosphatase activity and their observed sequence identities

Gene (accession no. of protein)	No. of encoded amino acids	Closest similar phosphatase protein, accession no. (no. of encoded amino acids), organism, E value	Identity to closest similar phosphatase protein (Blast), no. of amino acids similar/ total no. (%)	% identity to closest similar phosphatase protein (Clustal alignment)
*pho01* (AWN00218)	229	Phosphatidylglycerophosphatase, PIF15492.1 (224), *Rhodanobacter* sp. strain TND4EH1, 3E−99	161/213 (76)	72
*pho02* (AWN00219)^*a*^	339	Phosphoserine phosphatase, AFM25187 (342), Desulfomonile tiedjei DSM 6799, 0.0	251/337 (74)	74
*pho03A* (AWN00220)^*a*^	493	Phosphoesterase, WP_009239878.1 (404), *Ralstonia*, 2E−9	183/425 (49)	47
*pho03B* (AWN00221)^*b*^	222	Phospholipase/carboxylesterase, ADV48687.1 (334), Cellulophaga algicola DSM 14237, 2E−14	84/181 (46)	27
*pho04* (AWN00222)	214	Putative membrane-associated alkaline phosphatase, KGB26473 (203), Acetobacter tropicalis, 9E−50	92/193 (48)	46
*pho07* (AWN00223)^*a*^	392	Phosphoesterase family protein, PZS03611.1 (379), *Pseudonocardiales* bacterium, 1E−111	184/349 (53)	51
*pho08A* (AWN00224)^*a*^	235	Histidine phosphatase family protein, WP_074262886.1 (229), Paraburkholderia phenazinium, 2E−56	97/191 (51)	49
*pho08B* (AWN00225)^*a*^	236	Histidine phosphatase family protein, WP_090546752.1 (196), Paraburkholderia caballeronis, 1E−59	97/182 (53)	50
*pho08C* (AWN00226)^*a*^	238	Histidine phosphatase family protein, WP_090546752.1 (196), Paraburkholderia caballeronis, 2E−57	98/182 (54)	51
*pho09C* (AWN00227)^*a*^	455	Alkaline phosphatase family protein, WP_007415052.1 (407), Pedosphaera parvula, 0.0	330/413 (66)	63
*pho10* (AWN00228)	554	Mismatch repair ATPase, WP_014786775 (599), Terriglobus roseus, 6E−142	246/558 (44)	44
*pho13* (AWN00229)	411	Broad-specificity phosphatase PhoEn, WP_071949433.1 (401), *Mycobacterium* sp. strain PYR15, 0.0	349/400 (87)	83
*pho14A* (AWN00230)^*a*^^,^^*b*^	229	Protein tyrosine phosphatase (partial), CCZ50566.1 (64), *Acidobacteria* bacterium, 9E−13	43/111 (50)	48
*pho14B* (AWN00231)^*b*^	305	Phosphoserine phosphatase, PKM89459.1 (276), *Firmicutes* bacterium, 2E−4	58/215 (27)	25
*pho14C* (AWN00232)^*b*^	356	Phosphatidylserine/phosphatidyl glycerophosphate, AEQ20292 (371), uncultured bacterium CSLG7, 2E−109	175/357 (49)	48
*pho14D* (AWN00233)^*b*^	602	Protein tyrosine phosphatase, PYO70860.1 (581), *Gemmatimonadetes* bacterium, 1E−137	244/579 (42)	41
*pho15* (AWN00234)	223	Alkaline phosphatase, OFV86354.1 (209), *Acidobacteria* bacterium, 8E−34	71/167 (43)	41
*pho16A* (AWN00235)	819	Diguanylate cyclase/phosphodiesterase, WP_067501625.1 (816), *Actinoplanes* sp. strain TFC3, 1E−46	105/247 (43)	39
*pho16B* (AWN00236)^*a*^	376	Protein tyrosine phosphatase, WP_042381880.1 (372), Streptacidiphilus melanogenes, 0.0	257/324 (89)	76
*pho17A* (AWN00237)^*a*^	353	Phosphoserine phosphatase, AFM25187 (342), Desulfomonile tiedjei DSM 6799, 0.0	252/329 (77)	74
*pho18* (AWN00238)^*a*^	248	Phosphatase PAP2 family protein, WP_093286091.1 (257), *Verrucomicrobiaceae* bacterium GAS474, 4E−55	99/200 (50)	46
*pho19A* (AWN00239)	612	Alkaline phosphatase precursor, AMY11511 (577), *Acidobacteria* bacterium DSM 100886, 8E−126	230/529 (43)	42
*pho20B* (AWN00240)	392	Phosphoglycolate phosphatase, RDI59778.1 (337), Microvirga subterranea, 3E−152	248/339 (76)	73
*pho24* (AWN00241)	428	PAP2 superfamily protein, SHK15444 (414), Bradyrhizobium lablabi, 3E−141	215/405 (53)	54
*pho25B* (AWN00242)^*a*^^,^^*b*^	526	Phospholipase, WP_052891151 (505), Thermogemmatispora carboxidivorans, 0.0	303/527 (57)	60
*pho25C* (AWN00243)	252	Phospholipase, WP_006679394.1 (222), Paenibacillus dendritiformis, 0.0	41/101 (41)	28
*pho26* (AWN00244)^*a*^	559	Alkaline phosphatase family protein, WP_020714678.1 (564), *Acidobacteriaceae* bacterium KBS 89, 0.0	434/551 (79)	78
*pho27A* (AWN00245)^*a*^	347	Multispecies: phosphatase, WP_PYV87257.1 (338), *Acidobacteria* bacterium, 9E−64	249/323 (77)	74
*pho27B* (AWN00246)	263	Acid sugar phosphatase, GBD30013.1 (265), bacterium HR32, 2E−57	106/254 (42)	39
*pho28A* (AWN00247)^*b*^	490	Nonhemolytic phospholipase C, APW61637.1 (486), Paludisphaera borealis, 0.0	328/454 (72)	69
*pho28C* (AWN00248)^*a*^	232	Histidine phosphatase family protein, WP_106819986.1 (214), *Syntrophobacter* sp. strain SbD1, 2E−61	93/170 (53)	46

a Signal peptide detected.

b No phosphatase activity was detected on indicator plates after cloning ORF into expression vector.

From the 21 positive clones, seven harbored more than one putative phosphatase-related gene (Table 2). Thus, if two or more potential phosphatase activity-related genes were present in a positive clone, individual heterologous expression and subsequent phosphatase activity verification were performed. The analysis of colonies showed that the individual heterologous expression of 24 out of 31 genes led to phosphatase activity and the corresponding positive phenotype of the respective recombinant E. coli strains (Table 2).

### High phosphatase diversity recovered from soil metagenomes.

Phosphatases can be classified according to the structural fold of the catalytic domains and subclassified into families and subfamilies based on sequence similarities of the phosphatase domains, as well as by conserved amino acid motifs not belonging to the catalytic domain (6, 19). However, some are still classified based on their biochemical properties and biological functions (20).

Among the putative gene products encoded by the 31 candidate genes, alkaline phosphatases were identified as the most abundant group (five representatives), followed by histidine phosphatases and phospholipases with four representatives each. Phosphoserine-phosphatases and protein-tyrosine phosphatases were represented by three putative genes each. Acid phosphatases were encoded by two genes, while the plasmid pLP10 harbored an ORF with a deduced gene product showing similarity to a mismatch repair ATPase (Table 2).

**FIG 1:**
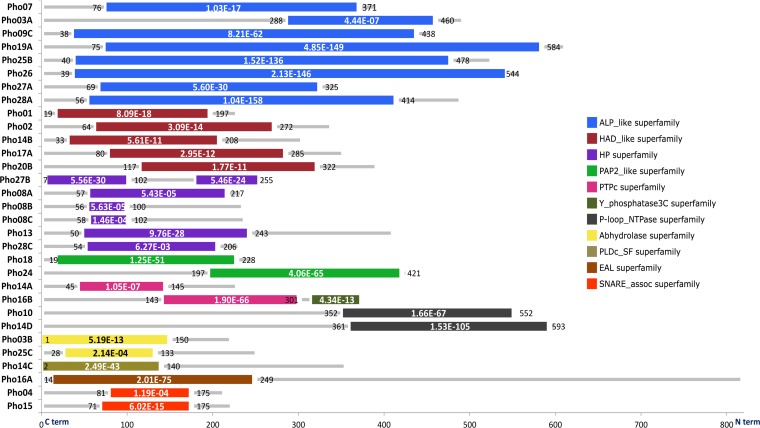
General architecture and domains of the retrieved phosphatases: ALP, alkaline phosphatases and sulfatases (cl23718); HAD, haloacid dehalogenase (cl21460); HP, histidine phosphatase (cl11399); PAP2, phosphatidic acid phosphatase (cl00474); PTPs, protein tyrosine phosphatases (cl21483); Y phosphatase 3C superfamily (cl6249); P-loop NTPase superfamily (cl21455); abhydrolase superfamily (cl21494); PLDc, phospholipase D (cl15239); EAL superfamily (cl00290); SNARE-associated superfamily (cl00429).

The amino acid sequence analysis revealed the presence of 10 different domains in the 31 deduced proteins. We detected the alkaline phosphatase and sulfatase superfamily domain (ALP-like cl23718) as the most frequent domain, represented in eight sequences. The second highest abundance showed the haloacid dehydrogenase domain (HAD cl21460), which was identified in six protein sequences. Three out of four classical phosphatase/phytase domains were detected in this study: the histidine phosphatase domain (HP with five protein sequences), the tyrosine phosphatase domain (PTPc with two protein sequences), and the acid phosphatase domain (PAP with two protein sequences) (Fig. 1). The phylogenetic analyses of the enzyme sequences and those harboring the above-mentioned domains revealed different clustering patterns in relation to reference phosphatase sequences for the different groups. Within the analyzed groups, the clustering of the metagenome-derived enzymes ranged from clear separation to integrated clustering (Fig. S2).

10.1128/mBio.01966-18.2 FIG S2 Phylogenetic trees of the retrieved phosphatases. Colors of each phylogenetic tree are consistent with the designated colors of the domains in Fig. 1. ALP-like (alkaline phosphatases and sulfatases cl23718): AGR55863.1, alkaline phytase, Bacillus subtilis; ADZ99372.1, beta-propellar phytase, *Bacillus* sp.; ABP02074.1, 3-phytase, Bacillus licheniformis; WP_046163127.1, alkaline phosphatase, *Pseudomonas*; WP_062274650.1, alkaline phosphatase, *Rhizobium*; HAD (haloacid dehalogenase superfamily cl21460): KZV11788.1, enzyme PHM8, Saccharomyces cerevisiae; WP_008764352.1, Cof-type HAD-IIB hydrolase, *Bacteroides*; GAX71936, glycerol-1-phosphatase, Saccharomyces cerevisiae; AAC43183.1, E-1 enzyme from Klebsiella oxytoca; ENZ46626, phosphatase, Clostridium bolteae; HP (histidine phosphatase cl11399): AEQ29498.1, histidine acid phytase, partial *Serratia* sp.; AEI69378.1, phytase, Yersinia mollaretii; XP_025481761.1, 3-phytase, Aspergillus neoniger; PAP2 (phosphatidic acid phosphatase cl00474): XP_015631975.1, purple acid phosphatase, Oryza sativa; ACR23331.1, purple acid phosphatase, Hordeum vulgare; ACR23326.1, purple acid phosphatase, Triticum aestivum; PTPs (protein tyrosine phosphatase cl21483): Q0SFJ4, possible tyrosine protein phosphatase, Rhodococcus jostii; WP_011797517.1, PTP, Acidovorax citrulli; P96830, PTP, Mycobacterium tuberculosis; ABC69367, protein tyrosine phosphatase-like inositol polyphosphate phosphatase, Selenomonas lacticifex; AAQ13669.1, myoinositol hexaphosphate phosphohydrolase, Selenomonas ruminantium; ABC69359.4, PTP-like phytase, Selenomonas ruminantium; P-loop_NTPase superfamily cl21455: AEP88384.1, protein YvcJ, Bacillus subtilis; Mrp protein, Dictyoglomus turgidum; CAA64779.1, Nbp35p protein, Saccharomyces cerevisiae; abhydrolase superfamily cl21494: LIP1_DIURU, Diutina rugosa; 1QE3_A, para-nitrobenzyl esterase, Bacillus subtilis; WP_014115247.1, carboxylesterase/lipase family protein, *Bacillus*; PLDc, phospholipase D cl15239: WP_109485171.1, phosphatidylserine synthase, Occallatibacter savannae; WP_011681875.1, phosphatidylserine/phosphatidylglycerophosphate/cardiolipin synthase-like, “*Candidatus* Solibacter usitatus”; WP_085199562.1, phospholipase, Mycobacterium fragae; WP_108063934.1, cardiolipin synthase, *Spartobacteria*; EAL superfamily cl00290: ACY88111.1, protein STM14_1632, Salmonella enterica; ABR76592.1, hypothetical protein KPN_01159, Klebsiella pneumoniae; AAP17004.1, hypothetical protein S1641, Shigella flexneri; AAK25358.1, GGDEF family protein, Caulobacter vibrioides; SNARE-associated superfamily cl00429: WP_005788593.1, DedA protein, *Bacteroides*; WP_025286307.1, DedA protein, Granulibacter bethesdensis; WP_118191286.1, DedA protein, Prevotella copri. Download FIG S2, PDF file, 1.1 MB. Copyright © 2019 Castillo Villamizar et al. 2019 Castillo Villamizar et al. This content is distributed under the terms of the Creative Commons Attribution 4.0 International license.

**FIG 2:**
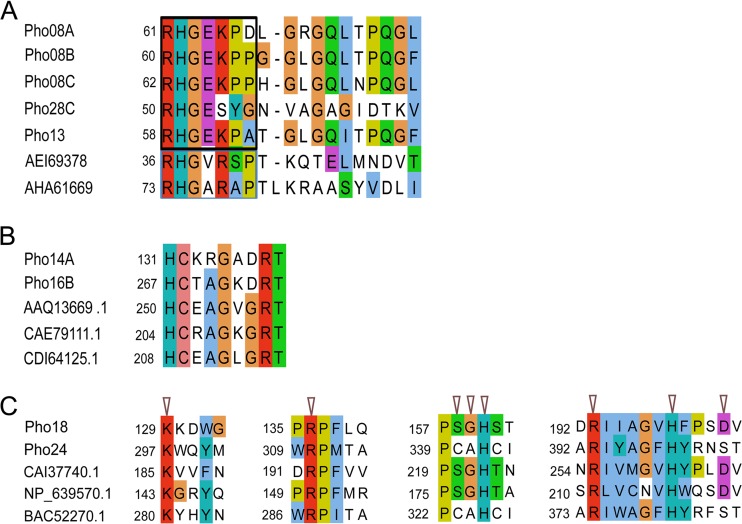
Multiple sequence alignments of conserved regions of phosphatases belonging to the HP, PTP, and PAP2 superfamily. (A) Blue line, typical conserved HP phytase motif (RHGXRXP) in AEI69378 (phytase from Yersinia mollaretii) and AHA61669 (histidine acid phosphatase phytase from Thermothelomyces thermophila). Black line, the variations of the motif found in this study. (B) Typical PTP motif (HCX5R) in Pho14A, Pho16B, AAQ13669 (myoinositol hexaphosphate phosphohydrolase from Selenomonas ruminantium), CAE79111 (protein tyrosine phosphatase 2 from Bdellovibrio bacteriovorus HD100), and CDI64125 (protein tyrosine phosphatase from Xylophilus ampelinus). (C) Catalytic sites of the PAP2 superfamily (cl00474), in Pho18, Pho24, CAI37740 (putative phosphatase from Corynebacterium jeikeium), NP_639570 (phosphatase from Xanthomonas campestris), and BAC52270 (phosphatase from Bradyrhizobium diazoefficiens).

The HP superfamily (cl11399) is represented by a diverse group of proteins divided into two branches exhibiting numerous functions (21). Classical members of the HAPhy share a conserved motif, RHGXRXP, characteristic for this enzyme class. The HAPhy catalytic reactions are based on the conserved histidine residue in the RHGXRXP motif (21, 22). In this study, all five phosphatases belonging to the HP superfamily harbored this histidine residue (Fig. 2A). Three out of five HPs in this survey were encoded by plasmid pLP08. The analysis of the plasmid sequence revealed a tandem organization of these genes with slight individual sequence variations (Fig. 2A; Fig. S3).

10.1128/mBio.01966-18.3 FIG S3 Insert of the plasmid pLP08 showing the tandem organization and relative position of the candidate genes *pho08A*, *-B*, and *-C*. Download FIG S3, PDF file, 0.1 MB. Copyright © 2019 Castillo Villamizar et al. 2019 Castillo Villamizar et al. This content is distributed under the terms of the Creative Commons Attribution 4.0 International license.

PTPs are well-studied proteins with a characteristic motif (HCX5R) (23, 24). In this study, two new PTPs (Pho14A and Pho16B) harboring the typical catalytic signature of the group (Fig. 2B) were detected. Interestingly, Pho16B showed the specific signature of the MptpB-like phosphatases characterized by the presence of the unique active site P-loop submotif HCXXXKDRT. This type of protein has been predicted in several microorganisms, including pathogens, but never in environmental samples. For the remaining group of classic phytases detected in this study (PAP), the literature describes two branches, the PAP1 enzymes, which are Mg^2+^-dependent enzymes, and the PAP2 enzymes, which are Mg^2+^ independent, but in all cases the active forms of PAP phytases were derived from plants (25). We detected the PAPs Pho18 and Pho24, which are affiliated with bacteria and belong to the Mg^2+^-independent branch (PAP2 cl00474) (Fig. 1 and 2C).

Alpha/beta hydrolases (abhydrolases) represent a group of proteins with a high number of substrates and catalytic functions (26). Two gene products (Pho03B and Pho25C) contained an abhydrolase domain (Fig. 1). However, only Pho25C showed phosphatase/phytase activity after individual heterologous expression of the corresponding gene. Abhydrolases exhibit broad substrate specificity, and some members have been reported with phospholipase activity (27).

Other ORFs such as Pho16A carry the EAL domain, which is present in diverse bacterial signaling proteins and encodes a phosphodiesterase function (28). Analysis of Pho10 and Pho14D amino acid sequences indicates the presence of the P-loop_NTPase superfamily domain (Fig. 1). Enzymes harboring this domain hydrolyze the beta-gamma phosphate bond of, e.g., ATP and GTP (29). In this study, Pho10 showed phosphatase activity, while Pho14D as part of the clone harboring plasmid pLP14 showed none. Pho14C showed no phosphatase activity after individual heterologous expression of the corresponding gene. The *pho14C* gene product harbors the phospholipase D catalytic domain (PLDC_SF domain) (30).

### SNARE-associated proteins with phosphatase activity harbor a new motif.

In 19 out of 21 positive clones, we identified at least one gene encoding a protein domain associated with catalytic activity of phosphatases. In contrast, the phosphatase-related genes of plasmids pLP04 and pLP15 did not encode known catalytic domains or signatures directly or indirectly associated with phosphatases. Clones carrying these plasmids showed significant phosphatase activity, and the products Pho04 and Pho15 showed sequence similarity to other previously reported proteins carrying the SNARE domain. However, both proteins shared overall sequence identity to previously reported phosphatases (Table 2). After individual heterologous expression of *pho04* and *pho15*, phosphatase activity was confirmed for both gene products. Pho04 and Pho15 hold the SNARE-associated domain DedA. SNARE-associated proteins are classified as structural proteins that function as a protein-protein interaction module (31). To our knowledge, no proteins with SNARE domains have been previously discovered to possess phosphatase activity.

**FIG 3:**
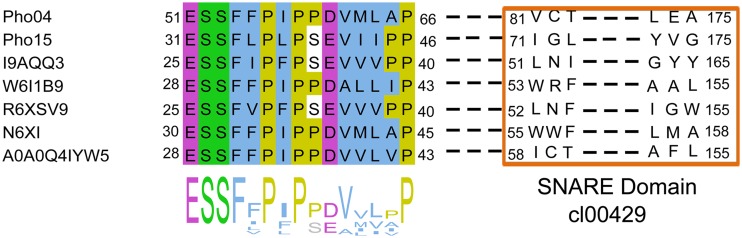
Partial multiple sequence alignment of Pho04, Pho15, and UniProt entries of SNARE-associated Golgi proteins. A detected conserved motif and its position in relation to the SNARE family are shown. The calculated consensus is depicted at the bottom. I9AQQ3, Bacteroides fragilis; W6I1B9, Granulibacter bethesdensis; R6XSV9, *Prevotella* sp.; N6XI35, *Thauera* sp.; and A0A0Q4IYW5, *Sphingomonas* sp.

We performed an alignment based on the *pho04* and *pho15* gene products, which revealed a shared conserved region (Fig. 3). Next, we analyzed all 56,539 sequences associated with the SNARE-associated Golgi proteins InterPro entry (IPR032816) with respect to motifs that were similar to those found in Pho04 and Pho15. A total of 905 sequences showed the conserved sequence pattern or a similar form. The sequence analysis revealed that Pho04 and Pho15 and the other 905 SNARE-associated (IPR032816) sequences share the particular amino acid arrangement ESSF(F/L/I/V)P. Notably, with respect to all analyzed proteins the identified motif was mostly from bacteria and detected outside the SNARE domain (cl00429) (examples are depicted in Fig. 3). Pho04 harbors the SNARE domain but shows 48% sequence identity to a putative membrane-associated alkaline phosphatase from Acetobacter tropicalis, while the closest phosphatase-related hit for Pho15 was an alkaline phosphatase from an *Acidobacteria* representative (43% identity) (Table 2).

### ALP-like superfamily and non-plant-derived PAP representatives showing phytase activity.

We selected the gene products of *pho07* and *pho18* for comprehensive biochemical characterization. The gene product of *pho07* does not contain any of the currently known catalytic domains associated with phytase activity. The only detected match of Pho07 was a nonspecific hit for the ALP-like superfamily (cl23718). In the case of *pho18*, the corresponding gene product comprises a domain of the purple acid phosphatases (PAP-like), which represents a type of phytase reported to be present in many organisms but is significantly expressed only in a very limited number of plant species (17, 32).

We successfully detected phytase activity of both purified enzymes, Pho07 and Pho18. Thus, to our knowledge Pho07 represents a new type of phytase and Pho18 represents the first PAP2 bacterial phytase. Furthermore, these two enzymes represent two out of the three reported environmental phytases derived from functional metagenomics. Both enzymes are putatively secreted by the natural bacterial host (Table S1) as the protein sequences harbor potential signal peptides of 30 (Pho07) and 22 (Pho18) amino acids at the N terminus. Pho07 shows the presence of an ALP-like superfamily domain (cl23718) (Fig. 1) and highest similarity to a phosphoesterase from a *Pseudonocardiales* representative (51% identity) (Table 2). Pho18 was most similar (50% identity) to an acid phosphatase from the *Verrucomicrobiaceae* member GAS474 (Table 2).

**FIG 4:**
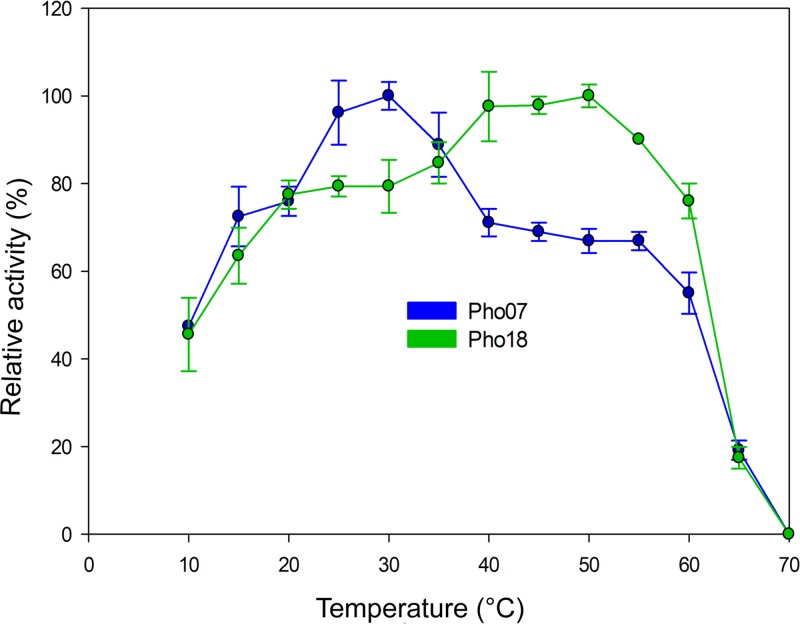
Effect of temperature on the relative activity of Pho07 and Pho18. All measurements were performed following the phytase standard assay at temperatures between 10 and 70°C. A 100% relative activity represented 2.9 and 1.04 U/mg for Pho07 and Pho18, respectively.

Pho07 and Pho18 exhibited optimal activity at 30 and 50°C, respectively (Fig. 4). After incubation of Pho07 for 4 h at 30°C, the enzyme retained more than 80% activity (Fig. S4). Incubation for 3 h at 45 and 60°C resulted in a substantial reduction (approximately 50%) and complete loss of enzyme activity, respectively. Pho18 retained approximately 80% activity after incubation for 6 h at 40°C but lost more than 50% of its activity at temperatures ≥50°C (Fig. S4).

10.1128/mBio.01966-18.4 FIG S4 Thermal stability of Pho07 (a) and Pho18 (b). All measurements were performed according to the phytase standard assay. Specific activities corresponding to 100% relative phytase activity are 3.14 (a) and 1.61 (b) U/mg. The average from triplicate experiments is presented. Download FIG S4, PDF file, 0.2 MB. Copyright © 2019 Castillo Villamizar et al. 2019 Castillo Villamizar et al. This content is distributed under the terms of the Creative Commons Attribution 4.0 International license.

**FIG 5:**
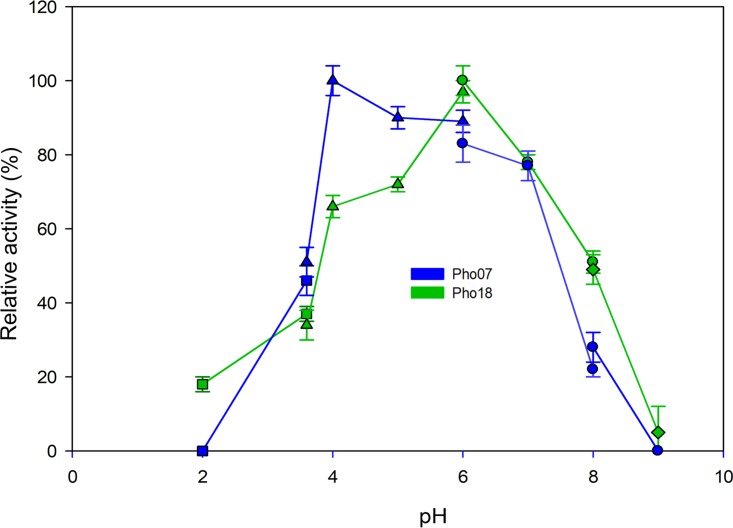
Effect of pH on the relative activity of Pho07 and Pho18. The measurements were performed with different buffer systems according to the phytase standard assay at the optimal temperature of each protein. The average from triplicate experiments is presented. Glycine-HCl buffer, squares; sodium acetate buffer, triangles; Tris-maleate buffer, circles; glycine-NaOH buffer, diamonds. 100% relative phytase activity represented 4.84 and 1.39 U/mg for Pho07 and Pho18, respectively.

**FIG 6:**
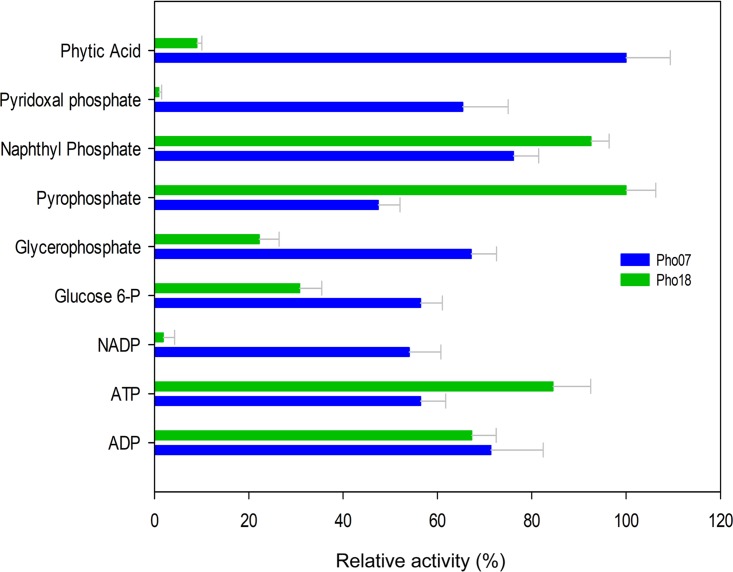
Substrate specificity of Pho07 and Pho18. Specific activities corresponding to 100% relative phytase and pyrophosphatase activities of Pho07 and Pho18 were 2.98 and 13.3 U/mg, respectively. All measurements were performed in triplicate and under optimal pH and temperature conditions for each enzyme.

We evaluated the optimal pH range using different buffer systems at 30°C for Pho07 and at 50°C for Pho18. Pho07 exhibited the highest activity at pH 4.0 (Fig. 5) and retained more than 80% of its activity between pH 5.0 and 7.0. Low or no enzymatic activity was detected at pH values lower than 2.0 and higher than 8.0. Pho18 showed the highest activity at pH 6.0 and retained more than 70% of its activity at pH 5.0 and 7.0 (Fig. 5). To determine the substrate specificity of Pho07 and Pho18, we tested several phosphorylated compounds as the substrates (Fig. 6). Pho07 released phosphate from all tested compounds with the highest activity toward phytate and lowest activity toward pyrophosphate. Pho18 showed the highest relative activity with pyrophosphate as the substrate and no significant activity with pyridoxal phosphate and NADP. As Pho07 and Pho18 exhibited the highest activity with phytate and pyrophosphate, respectively, we used these substrates for calculation of kinetic constants (Table 3).

**TABLE 3:** Kinetic values of Pho07 and Pho18 under optimal pH and temperature conditions

Enzyme	Mean (3 expts) ± SD							
	*K_m_* (mM)		*V*_max_ (μmol min^−1^ mg^−1^)		*k*_cat_ (min^−1^)		*k*_cat_/*K_m_* (min^−1^ M^−1^)	
	Sodium phytate	Pyrophosphate	Sodium phytate	Pyrophosphate	Sodium phytate	Pyrophosphate	Sodium phytate	Pyrophosphate
Pho07	0.49 ± 0.18	1.09 ± 0.03	6.50E−03 ± 1.01E−06	1.30E−04 ± 8.05E−06	694 ± 12.43	516 ± 22.98	3,410 ± 122	4,991 ± 155
Pho18	0.96 ± 0.09	0.22 ± 0.04	2.82E−03 ± 2.01E−04	4.03E−04 ± 4.42E−07	152 ± 9.83	1,088 ± 34.09	1,550 ± 18	49,200 ± 274

**FIG 7:**
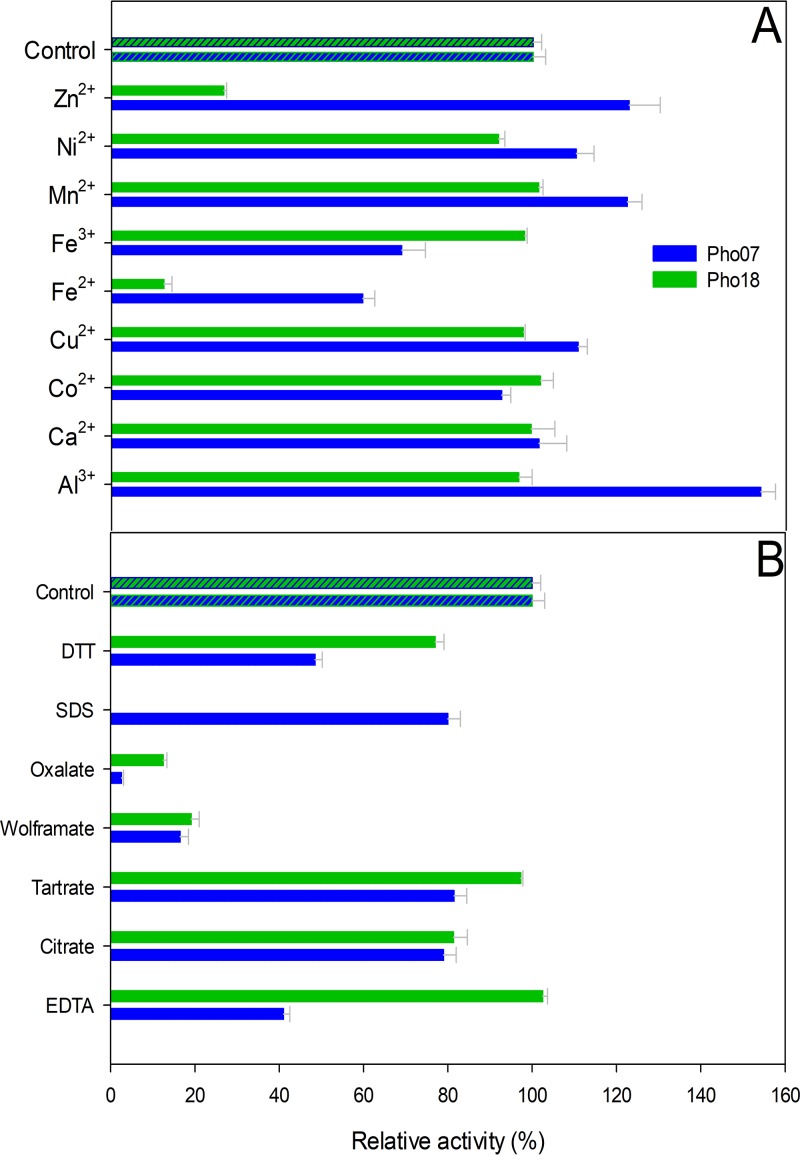
Effect of (A) metal ions and (B) potential inhibitors at 1 mM on the relative activity of Pho07 and Pho18. Specific activity values expressed as percentages of the control reactions are 3.8 and 1.3 U/mg for Pho07 and Pho18, respectively (A), and 3.5 and 1.22 U/mg for Pho07 and Pho18 (B), respectively.

Finally, we measured the effect of various metal ions and potential enzyme inhibitors on the activity of Pho07 and Pho18 with phytate as the substrate (Fig. 7). The metal ions showed different effects on the activity of the analyzed proteins. Al^3+^, Mn^2+^, and Zn^2+^ increased the activity of Pho07, while the activity of Pho18 decreased in the presence of Zn^2+^. Fe^2+^ had a strong inhibitory effect on the activity of both enzymes. With respect to potential inhibitors, the strongest inhibitory effects were observed at concentrations of 1 mM. Pho07 and Pho18 activities were reduced by most of the tested inhibitors. Oxalate was the strongest inhibitor for Pho07, while the activity of Pho18 was completely depleted in the presence of SDS (Fig. 7).

## DISCUSSION

Apart from the relationship with pathogenesis and the economic relevance, phosphatases/phytases are also important for reduction of phosphorus pollution and its impact on diverse environments (8, 11, 13). However, only a few phosphatases, most of them from cultivable organisms, have been comprehensively analyzed. The discovery of new phosphatases from environmental samples as well as engineering of available representatives of this enzyme group is considered a major research challenge (33). So far, few studies have attempted to discover phosphatases/phytases encoded by metagenomes using a function-based approach. Within these studies, only three genes and one of the corresponding proteins which exhibited phytase activity were recovered and described (34–36). We found 31 candidate genes, and 24 of them encoded phosphatase activity after individual heterologous expression (Table 2). For the remaining seven genes, activity was not detected at individual gene level. The corresponding gene products might be part of larger phosphatase units or require other components encoded by the insert to show phosphatase activity.

Approximately 55% of the gene products described in this study showed low protein sequence identity to known phosphatases (50% or less) (Table 2), which demonstrates the capacity of our screening method to identify novel enzymes with phosphatase activity from environmental samples. It has been previously discovered that the absence of free phosphate and the addition of phytate to medium induce the expression of phytases (37). Therefore, it is indicated that many of the detected genes encode new enzymes with phytase activity as observed for Pho07 and Pho18.

ALP phosphomonoesterases widely occur in nature. They preferably hydrolyze phosphate esters at pH levels higher than 7.0 (38). The ALP-like superfamily (cl23718) was the most abundant domain we detected in the recovered hits derived from our soil metagenomic libraries. The pH of the soil samples used ranged from 3.1 to 4.5 (39). Nevertheless, acid phosphatase genes are considered to be more abundant than alkaline phosphatase genes in low-pH soils. This might be due to the fact that most studies on the prevalence of alkaline and acid phosphatase genes are based on PCR-based gene amplification using specific known genes from cultured individual species as starting point for primer design (40). This approach covers only a small fraction of the existent functional phosphatase genes. Here, we revealed the existence of so-far-unknown functional ALPs with low identity toward known phosphatases, evidencing the potential of our functional metagenomic approach for the discovery of new ALP-phosphatases from environmental samples.

To our knowledge, enzymes from the ALP-like superfamily entry (cl23718) exhibiting phytase activity have not been described or comprehensively characterized yet. Nevertheless, numerous proteins are mentioned in literature or annotated in databases as alkaline phosphatases with phytase activity, but their molecular signatures and domains are associated mostly with the classic phytases (14). The analysis by Lim et al. (41) focusing on the distribution and diversity of phytate-mineralizing bacteria considers alkaline phosphatases to be ubiquitous in living organisms and shows that they dephosphorylate a wide range of P compounds, but not phytate. Thus, the functional proteins carrying the ALP-like superfamily domain reported in this study (7) represent a new group of phytase enzymes. The phylogenetic analysis of the ALP-like members revealed that most of our metagenome-derived enzymes cluster separately from previously reported alkaline phosphatases/phytases (see Fig. S2 in the supplemental material).

The biochemical analysis of a selected ALP-like member, Pho07, showed that its temperature optimum is similar to the metagenome-derived alkaline phosphatase (mAP). This enzyme is one of the few reported phosphatases derived from environmental samples and not associated with cultures (42). Furthermore, the optimal pH range of Pho07 (4.0 to 5.0) is similar to that of other soil bacterial phytases (43). Among the tested substrates, Pho07 showed the highest activity toward phytate, indicating that its primary activity is related to the degradation of this compound. Several studies report an enhancing effect of Ca^2+^ and Mn^2+^ on phytase activity (43). Nevertheless, the activity of Pho07 increased in the presence of Mn^2+^, but it was not affected by Ca^2+^. Among the potential inhibitors, wolframate and oxalate did not show significant effects on the activity of a phytate-degrading enzyme from Pantoea agglomerans (44) but reduced the relative activity of Pho07 to values lower than 20%. Since Pho07 is the first reported phytase carrying an ALP-like domain, it is not possible to compare its kinetic parameters (Table 3) with those from phytases of the same type.

The enzyme Pho18 belongs to the known PAPphy group of phytases. Only a few examples of characterized PAP proteins with phytase activity have been previously reported, and all of them were derived from plants (25). However, the presence of PAP-related genes in mammals, fungi, and bacteria has been indicated based on annotated genome sequences. The taxonomic analysis of *pho18* and the complete insert harboring it revealed a bacterial origin and a phylogenetic association with the genus *Terrimicrobium* of the *Verrucomicrobia* phylum (Table S1). In addition, biochemical analysis confirmed phytase activity of Pho18. Therefore, we report here for the first time a PAP2 phosphatase with phytase activity, which is of nonplant origin and metagenome derived. Moreover, the phylogenetic analysis showed that Pho18 clusters separately from other previously reported PAPs with phytase activity. The reason for this is most likely the vegetal origin of the previously reported PAP phytases (Fig. S2). To our knowledge, the study of Ghorbani Nasrabadi et al. (45) is the only attempt to identify PAP phytases derived from bacteria. In their study, an indirect association between phytase activity and the amplification of a putative PAP gene in the bacterial host was established (45).

The optimal temperature of Pho18 (50°C) is similar to optimal temperatures of other PAPs derived from wheat (45°C) and soybeans (58°C) (14). Furthermore, the behavior of Pho18 at temperatures higher than 55°C (Fig. 4) is similar to that reported for soybean phytases (46). An increase of phytase activity mediated by the addition of Mn^2+^ was reported for PAP phytases (32, 43). We did not register significant increases in the activity of Pho18 in the presence of any cation. However, the enzyme was strongly inhibited by Zn^2+^, which is in contrast to other PAP phytases showing higher activity in the presence of this ion. Although Pho18 exhibits higher affinity to pyrophosphate, the kinetic parameters using phytate as the substrate are similar to PAP phytases from *Arabidopsis* (Table 3) (47).

We found the HAD (cl21460) domain as the second most abundant domain in our survey. The HAD domain is present in proteins of diverse organisms, including bacteria, archaea, and eukaryotes (48). This domain is carried by proteins able to catalyze a variety of biological functions and act on a wide range of substrates (19). Numerous members of the HAD superfamily can transfer phosphoryl groups or act as phosphoanhydride hydrolase P-type ATPases (49). Since proteins harboring this domain are involved in a variety of cellular processes, it is not surprising that they can be isolated through functional metagenomic screening for phosphatases.

One of the most remarkable findings in this study was the detection of the SNARE-associated domain (DedA, InterPro entry IPR032816) of Pho04 and Pho15. So far, the role of the SNARE-associated domain (DedA) has not been deeply studied. Bacterial DedA family mutants display phenotypes evidencing cell division defects, temperature sensitivity, and altered membrane phospholipid composition among others (50). DedA-SNAREs have been reported to promote or block membrane fusion, particularly during bacterial pathogenic processes (51). To our knowledge no phosphatase activity has been reported for proteins harboring SNARE-associated domains. Moreover, the particular signature ESSF(F/L/I/V)P has been overlooked until now.

In conclusion, we demonstrate here for the first time the potential of functional metagenomics to exploit the phosphatase pools hidden in environmental samples. Our study revealed new phosphatases/phytases with diverse and, so far, largely unknown characteristics. Furthermore, we discovered the existence of a new type of phytases (ALP-like-phy) and found that the classical PAPphy are also functional in microorganisms and not only in plants.

## MATERIALS AND METHODS

### Soil sampling, DNA extraction, and construction of metagenomic libraries.

Genes encoding phosphatases were recovered from metagenomic libraries derived from A horizons of soil samples, which had been taken from forest sites of the German Biodiversity Exploratories Schwäbische Alb (samples AEW1 and AEW5), Hainich-Dün (sample HEW30), and Schorfheide-Chorin (samples SEW2, SEW5, and SEW46). Collection of samples was performed previously as described by Kaiser et al. (52) and Nacke et al. (39), respectively. Soil characteristics are available in Nacke et al. (39). Names of constructed metagenomic libraries refer to the designation of the samples from which the libraries were derived. Metagenomic libraries were generated using the method described by Nacke et al. (39). The plasmid libraries AEW1, AEW5, SEW2, and SEW5 have been previously generated by employing the same approach (39).

### Function-based screening and identification of ORFs encoding phosphatase activity.

For function-based screening of metagenomic libraries, we used our recently described method (18). Small‐insert libraries were constructed using the plasmid pCR-XL-TOPO as vector (Invitrogen GmbH, Karlsruhe, Germany) and Escherichia coli DH5α [F^–^ φ80*lacZ*Δ*M15* Δ(*lacZYA*-*argF*)*U169 recA1 endA1 hsdR17*(r_K_^–^ m_K_^+^) *phoA supE44* λ^–^ *thi*-*1 gyrA96 relA1*] as screening host. Modified Sperber medium (16 g/liter agar, 10 g/liter glucose, 500 mg/liter yeast extract, 100 mg/liter CaCl_2_, and 250 mg/liter MgSO_4_) was used a screening medium supplemented with 2.5 g/liter phytic acid as sole P source and 25 µg/ml of 5-bromo-4-chloro-3-indolyl phosphate (BCIP) (53). The modified Sperber minimal medium used in this study was used for detection of phosphatase/phytase activity of the library-bearing E. coli clones due to the presence of phytate and the absence of other inorganic P sources (37). The slight background activity observed after more than 48 h of incubation of the host strain is probably caused by the alkaline phosphatase-encoding gene (*phoA*) of the host. Positive clones show an intense dark blue colony color, whereas negative colonies exhibit first a white and subsequently a light blue or green color after prolonged incubation.

The plasmids derived from positive clones were sequenced by the Göttingen Genomics Laboratory (Göttingen, Germany), and ORF prediction was performed as described by Nacke et al. (39). Next, the obtained sequences were analyzed by using the Basic Local Alignment Search Tool (BLAST) (54). Only plasmids harboring at least one ORF potentially associated with phosphatase activity were considered candidates for further analysis and designated pLP01 to pLP04, pLP07 to pLP10, pLP13 to pLP20, and pLP24 to pLP28. Full-length sequence alignment was performed between the candidates and their closest related sequence by using Clustal Omega (55). All coding sequences were examined for similarities to known protein families and domains by performing searches against the InterPro collection of protein signature databases and conserved domain databases (CDD) (56, 57). The prediction of signal peptides of the proteins was performed by using SignalP 4.0 (58). Additionally, all inserts were taxonomically classified by using the software Kaiju 1.5.0 (59). Alignments of the deduced protein sequences and phylogenetic trees of the proteins were performed by using MEGA 7 (60). The maximum likelihood method based on the equal input model was applied. The bootstrap values were calculated from 500 replicates, and branches corresponding to partitions reproduced in fewer than 50% of bootstrap replicates were collapsed. Alignments were visualized by using Jalview version 2 (61).

Candidate genes encoding domains that have not been previously associated with phosphatase activity (pLP04 and pLP15) and inserts comprising more than one potential phosphatase-encoding gene were amplified and subsequently cloned. Specific primers for each target gene were designed, and the pET101/D directional TOPO cloning kit was used for cloning as recommended by the manufacturer (Thermo Fisher Scientific GmbH, Schwerte, Germany). PCR was carried out in a 50-μl volume containing 10 μl of 5-fold Phusion GC buffer, 200 μM (each) dNTP, 1.5 mM MgCl_2_, 2 μM (each) primers, 2.5% DMSO, 0.5 U Phusion High Fidelity Hot Start DNA polymerase (Thermo Fisher Scientific GmbH, Schwerte, Germany), and 25 ng recombinant plasmid. PCR conditions were as follows: initial denaturation at 98°C for 2 min followed by 30 cycles of denaturation at 98°C for 1.5 min, annealing at 58°C for 1 min, and extension at 72°C for 1 min, followed by a final extension at 72°C for 5 min. Subsequently, the amplified genes were individually cloned into the expression vector pET 101/D and transformed into E. coli BL21 [F^–^ *ompT hsdS*_B_(r_B_^–^ m_B_^–^) *gal dcm* (DE3)] as recommended by the manufacturer (Thermo Fisher Scientific GmbH). The resulting recombinant plasmid-bearing E. coli BL21 strains were subsequently plated on Sperber minimal medium agar supplemented with phytic acid (2.5 g/liter), BCIP (25 mg/ml), and IPTG (0.25 mM) for phosphatase activity detection.

### Heterologous expression of *pho07* and *pho18* and purification of gene products.

The genes *pho07* and *pho18* carried by plasmids pLP07 and pLP18, respectively, were selected for heterologous expression with the pET-20b (+) (V5-epitope/His tag) vector (Merck KGaA, Darmstadt, Germany) as recommended by Villamizar et al. for metagenome-derived phosphatases (18). Crude extracts containing the target proteins were derived from the expression strain E. coli BL21 and filtered as described by Villamizar et al. (18). For purification of the proteins, the filtered crude extracts were then transferred to nickel columns (Protino2000 Ni-Ted columns; Macherey and Nagel, Düren, Germany). The equilibration of the columns and the washing steps were performed with 50 mM HEPES buffer (pH 8.0) containing 200 mM NaCl, followed by three elution steps with 50 mM HEPES, 200 mM NaCl, and 250 mM imidazole. Pho07 was further purified by using the Äkta FPLC system (GE Healthcare, Little Chalfont, United Kingdom) via hydrophobic interaction chromatography. A 15PHE 4.6/100PE Tricorn high-performance column (GE Healthcare, Little Chalfont, United Kingdom) in a total bed volume of 1.7 ml with a 2-ml/min flow rate at room temperature was utilized. Pho18 was purified through ion-exchange chromatography, by using a cation exchanger (SOURCE15S) in a prepacked Tricorn column (4.6/100 PE) (GE Healthcare, Little Chalfont, United Kingdom) with a gel bed volume of 1.7 ml at a 1-ml/min flow rate and room temperature. The purity of the resulting protein preparations was analyzed by sodium dodecyl sulfate-polyacrylamide gel electrophoresis (SDS-PAGE), and the detection of V5 epitope-carrying proteins was achieved by Western blot hybridization, as described by Waschkowitz et al. (62).

### Enzyme assays.

Phosphatase activity was determined at 355 nm by detecting the release of inorganic phosphorus according to the ammonium molybdate method developed by Heinonen and Lahti with modifications (44, 63) as follows: the enzyme solutions (10 μl) were preincubated for 3 min at 40°C in 380 μl of 50 mM sodium acetate buffer (pH 5). Subsequently, 10 μl of 100 mM phytic acid dipotassium salt (Sigma-Aldrich, Munich, Germany) was added, and the mixture was incubated for 30 min at 40°C. To stop the reaction, 1.5 ml of freshly prepared AAM solution (acetone–5 N H_2_SO_4_–10 mM ammonium molybdate) and 100 μl of 1 M citric acid were added. Blanks were prepared by adding AAM solution prior to the addition of enzyme. The absorbance (355 nm) was measured using the Ultrospec 3300 Pro (Amersham plc, Little Chalfont, United Kingdom).

To assess the influence of pH on purified enzymes, the activity was measured at 40°C in a pH range from 1 to 9. The following overlapping buffer systems were used: 50 mM glycine-HCl (pH 1.0 to 3.5), 50 mM sodium acetate (pH 3.5 to 6.0), 50 mM Tris-maleate acid (pH 6.0 to 8.0), and 50 mM glycine-NaOH (pH 7.0 to 9.0). After the optimal pH was determined for Pho07 and Pho18, the influence of temperature on enzymatic activity was analyzed. The thermal stability was checked after incubation of the purified enzymes at different temperatures.

The substrate specificity of the phosphatases was determined using the standard assay described above under the optimal temperature and pH for each enzyme (substrate concentration, 10 mM). Furthermore, the effects of cations (Al^3+^, Ca^2+^, Co^2+^, Fe^2+^, Fe^3+^, Mn^2+^, Ni^2+^, and Zn^2+^) and the potential inhibitors (EDTA, citrate, tartrate, wolframate, oxalate, sodium dodecyl sulfate (SDS), and dithiothreitol (DTT) at concentrations of 0.1 and 1 mM were analyzed.

For the kinetic constants, all measurements were performed in triplicate under optimal pH and temperature conditions using phytic acid and pyrophosphate as the substrates. The data were analyzed by the Sigma Plot Enzyme Kinetic Module version SigmaPlot 12.0 (Systat Software, Inc., San Jose, CA).

### Sequence accession numbers.

The nucleotide sequences of plasmids listed in Table 1 have been submitted to the National Center for Biotechnology Information (NCBI) GenBank database under the accession numbers indicated: pLP01 (Pho01), KY931670; pLP02 (Pho02), KY931671; pLP03 (Pho03A and -B), KY931672; pLP04 (Pho04), KY931673; pLP07 (Pho07), KY931674; pLP08 (Pho08A to -C), KY931675; pLP09 (Pho09C), KY931676; pLP10 (Pho10), KY931677; pLP13 (Pho13), KY931678; pLP14 (Pho14A to -D), KY931679; pLP15 (Pho15), KY931680; pLP16 (Pho16A and -B), KY931681; pLP17 (Pho17A), KY931682; pLP18 (Pho18), KY931683; pLP19 (Pho19A), KY931684; pLP20 (Pho20B), KY931685; pLP24 (Pho24), KY931686; pLP25 (Pho25B and -C), KY931687; pLP26 (Pho26), KY931688; pLP27 (Pho27A and -B), KY931689; and pLP28 (Pho28A and Pho28C), KY931690.
