# Supplementary figures and images for: Functional Metagenomics Reveals an Overlooked Diversity and Novel Features of Soil-Derived Bacterial Phosphatases and Phytases
Source: mBio. 2019 Jan 29;10(1):e01966-18. doi: 10.1128/mBio.01966-18 (PMC6355987; doi:10.1128/mBio.01966-18)

**Negative Control**

**Candidate  
pLP03**

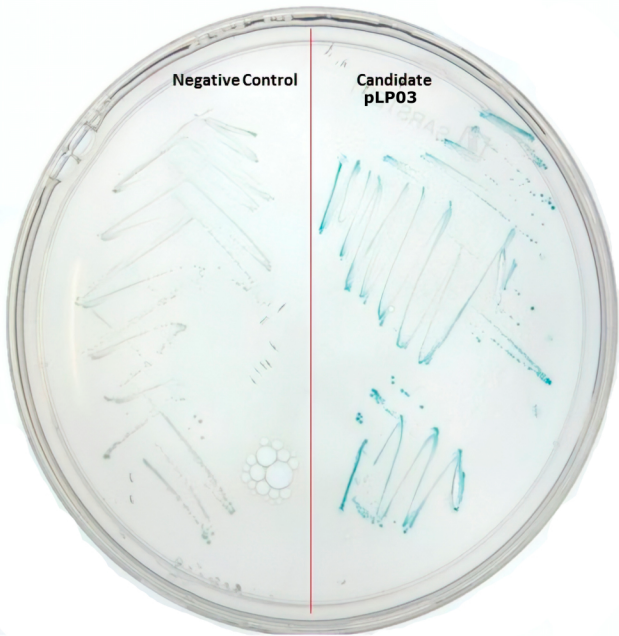

Supplement: FIG S1 [file mBio.01966-18-sf001.pdf]

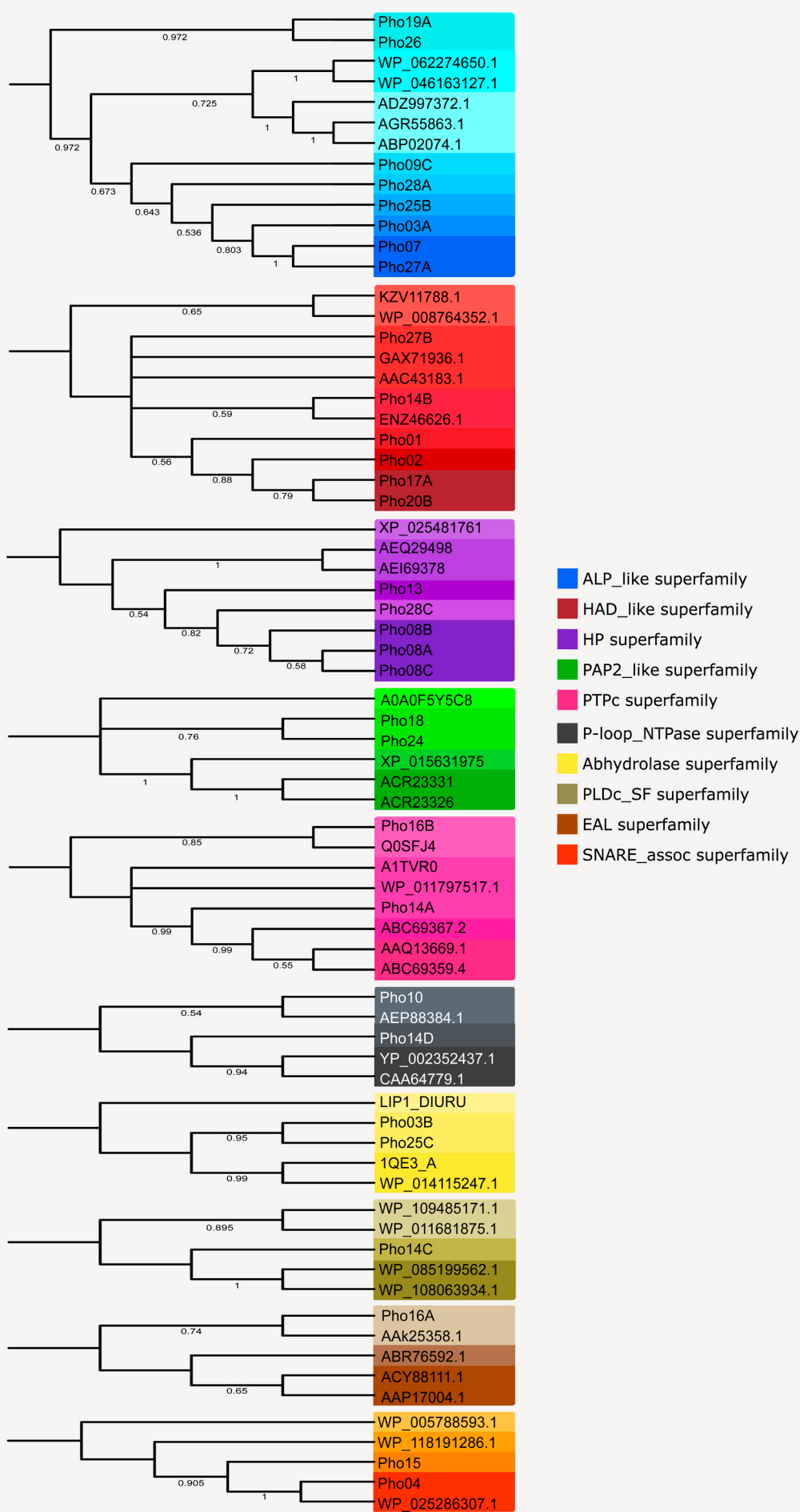

Supplement: FIG S2 [file mBio.01966-18-sf002.pdf]

pLP08 in pCR-XL-TOPO (9,960 bp)

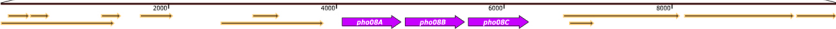

Supplement: FIG S3 [file mBio.01966-18-sf003.pdf]

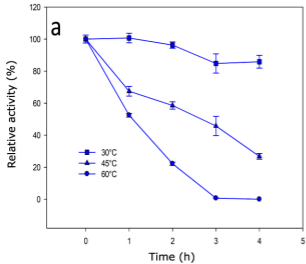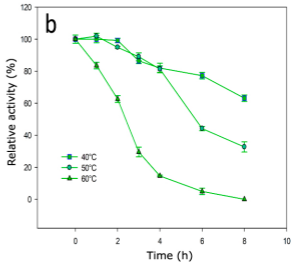

Supplement: FIG S4 [file mBio.01966-18-sf004.pdf]
