# Supplementary material for: Functional Metagenomics Reveals an Overlooked Diversity and Novel Features of Soil-Derived Bacterial Phosphatases and Phytases
Source: mBio. 2019 Jan 29;10(1):e01966-18. doi: 10.1128/mBio.01966-18 (PMC6355987; doi:10.1128/mBio.01966-18)
Supplement: TABLE S1 [file mBio.01966-18-st001.pdf]

Table S1. Taxonomic classification of inserts from the positive clones harboring phosphatase-related genes by using KAIJU 1.5.0

Plasmid	Taxonomic classification of insert
pLP01	Cellular organisms; Bacteria; Proteobacteria; Gammaproteobacteria; Xanthomonadales; Rhodanobacteraceae; Rhodanobacter; *Rhodanobacter* sp. C03
pLP02	Cellular organisms; Bacteria; environmental samples; uncultured bacterium
pLP03	Cellular organisms; Bacteria; Terrabacteria group; Actinobacteria; Actinobacteria; Streptomycetales; Streptomycetaceae; Streptacidiphilus; *Streptacidiphilus jiangxiensis*
pLP04	Cellular organisms; Bacteria; Proteobacteria; Alphaproteobacteria; Caulobacterales; unclassified Caulobacterales; Caulobacterales *bacterium* RIFOXYB1_FULL_67_16
pLP07	Cellular organisms; Bacteria; Terrabacteria group
pLP08	Cellular organisms; Bacteria; Proteobacteria; Oligoflexia; Bdellovibrionales
pLP09	Cellular organisms; Bacteria; PVC group; Verrucomicrobia; unclassified Verrucomicrobia; unclassified Verrucomicrobia (miscellaneous); Verrucomicrobia *bacterium* RIFCSPLOWO2_12_FULL_64_8
pLP10	Cellular organisms; Bacteria
pLP13	Cellular organisms; Bacteria; Terrabacteria group; Actinobacteria; Actinobacteria; Corynebacteriales; Mycobacteriaceae; Mycobacterium; environmental samples; uncultured *Mycobacterium* sp.
pLP14	Cellular organisms; Bacteria; Terrabacteria group; Chloroflexi; unclassified Chloroflexi; unclassified Chloroflexi (miscellaneous); *Chloroflexi bacterium* 13_1_40CM_55_7
pLP15	Cellular organisms; Bacteria; PVC group; Verrucomicrobia; Opitutae; unclassified Opitutae; Opitutae *bacterium* TMED102
pLP16	Cellular organisms; Bacteria; Terrabacteria group; Actinobacteria; Actinobacteria; Streptomycetales; Streptomycetaceae; Streptacidiphilus; *Streptacidiphilus melanogenes*
pLP17	Cellular organisms; Bacteria; Proteobacteria; Alphaproteobacteria; Rhizobiales; Bradyrhizobiaceae; *Bradyrhizobium*
pLP18	Cellular organisms; Bacteria; PVC group; Verrucomicrobia; Spartobacteria; Terrimicrobium; *Terrimicrobium sacchariphilum*;
pLP19	Cellular organisms; Bacteria
pLP20	Cellular organisms; Bacteria; Proteobacteria; Alphaproteobacteria; Rhizobiales; Bradyrhizobiaceae; Bradyrhizobium; *Bradyrhizobium paxllaeri*
pLP24	Cellular organisms; Bacteria; Proteobacteria; Alphaproteobacteria; Rhizobiales; Bradyrhizobiaceae; Bradyrhizobium; *Bradyrhizobium* sp. AS23.2
pLP25	Cellular organisms; Bacteria; PVC group; Verrucomicrobia; unclassified Verrucomicrobia; unclassified Verrucomicrobia (miscellaneous)
pLP26	Cellular organisms; Bacteria; Acidobacteria; Acidobacteriia; Acidobacteriales; Acidobacteriaceae; unclassified Acidobacteriaceae; Acidobacteriaceae *bacterium* KBS 89
pLP27	Cellular organisms; Bacteria; Terrabacteria group; Chloroflexi; Ktedonobacteria; Ktedonobacterales; Ktedonobacteraceae; *Ktedonobacter*
pLP28	Cellular organisms; Bacteria; Terrabacteria group; Actinobacteria; Actinobacteria; Streptomycetales; Streptomycetaceae; Streptomyces; *Streptomyces griseoplanus*
